# Immune Checkpoint Inhibitor‐Related Dysautonomia in Merkel Cell Carcinoma: A Case Report

**DOI:** 10.1002/cnr2.70274

**Published:** 2025-07-07

**Authors:** Nidhi Kuchimanchi, Sai Gajula, Elizabeth M. Gaughan, Russell G. Witt

**Affiliations:** ^1^ School of Medicine University of Virginia Charlottesville Virginia USA

**Keywords:** case report, dysautonomia, immune checkpoint inhibitor, immune‐related adverse event, nivolumab

## Abstract

**Background:**

Immune checkpoint inhibitors (ICIs) are monoclonal antibodies that block inhibitory pathways that cancer cells exploit to suppress T‐cell activation. Although immune‐related adverse events (irAEs) linked to ICI therapy are well documented and encompass dermatologic, endocrine, gastrointestinal, hepatic, and neurologic systems, ICI‐related dysautonomia remains a rare phenomenon. Management of ICI‐related dysautonomia is undefined.

**Case:**

We report the case of a 57‐year‐old male patient treated with neoadjuvant nivolumab for Merkel cell carcinoma who developed ICI‐related dysautonomia. His dysautonomia was characterized by orthostatic hypotension, urinary retention, hearing loss, and binocular diplopia in addition to the development of ICI‐related hepatitis. We describe the patient's course, including the treatment and outcome of his dysautonomia, and review the literature on this rare toxicity.

**Conclusion:**

Due to the mechanism of action of ICIs, irAEs can present with a wide range of manifestations. In this case, prompt recognition of ICI‐induced dysautonomia and timely administration of intravenous immunoglobulin (IVIG) led to significant clinical improvement. ICI‐induced dysautonomia is a rare condition that is difficult to diagnose and manage.

**Abbreviations**
AAG: autoimmune autonomic ganglionopathy
CAM: anti‐cytokeratin
CD: cluster of differentiation
CK: cytokeratin
CTLA: cytotoxic T‐lymphocyte antigen
ESMO: European Society for Medical Oncology
gAChR: ganglionic nicotinic acetylcholine receptor
GERD: gastroesophageal reflux disease
ICIs: immune checkpoint inhibitors
irAEs: immune‐related adverse events
IVIG: intravenous immunoglobulin
LAG: lymphocytes activation gene
MCC: merkel cell carcinoma
PD: programmed death
PDL: programmed death ligand

## 1 Introduction

Merkel cell carcinoma (MCC) is a rare neuroendocrine neoplasm that occurs on sun‐exposed areas of the skin. Risk factors for MCC include the male sex, increasing age, infection with Merkel cell polyomavirus, immunosuppression, UV radiation, and a prior diagnosis of malignancy [1]. Malignancies associated with MCC include melanoma, multiple myeloma, chronic lymphocytic leukemia, and non‐Hodgkin lymphoma [2]. First‐line treatment of MCC is surgical resection. Prior to the emergence of immunotherapy, tumors that were not amenable to surgery were treated with radiation and/or chemotherapy. However, MCC quickly developed resistance to chemotherapeutic regimens [3]. Subsequent results of phase II immunotherapy trials led to the approval of the immune checkpoint inhibitors (ICIs), pembrolizumab for locally advanced/metastatic MCC not amenable to definitive surgery or radiotherapy, and avelumab for metastatic MCC [4, 5]. The CheckMate 358 trial evaluated the safety and efficacy of neoadjuvant nivolumab, an anti‐programmed death‐1 immunotherapy, in patients with stage IIA–IV MCC. Results from the trial showed that 47.2% of patients with high‐risk resectable MCC who received neoadjuvant nivolumab achieved complete response. Additionally, among the responders, there was significant improvement in recurrence‐free survival. Together, these findings indicated that neoadjuvant nivolumab could be used as an adjunct to surgical resection [6].

ICIs are monoclonal antibodies that inhibit the regulation of T‐cell activation. Side effects, known as immune‐related adverse events (irAEs), mimic autoimmune diseases. Common irAEs are dermatologic, endocrine, gastrointestinal, and hepatic toxicities [7]. Risk factors, type, and severity of irAEs are difficult to predict on a patient‐to‐patient basis [8, 9]. While many of these toxicities have been well characterized, ICI‐related dysautonomia is rarely documented in literature. Thus far, in previously published cases of ICI‐related dysfunction of the autonomic nervous system, the most common symptoms of dysautonomia are orthostatic hypotension, urinary retention, and gastrointestinal dysfunction [10]. However, diagnosing patients with ICI‐related dysautonomia has proven to be challenging due to its resemblance to other irAEs, such as autoimmune adrenal insufficiency [11], Here, we report the case of a 57‐year‐old man undergoing neoadjuvant nivolumab treatment for MCC who developed ICI‐related dysautonomia, hepatitis, and diplopia. We aim to further characterize and discuss treatment options for ICI‐related dysautonomia to provide clarity to this challenging diagnosis and increase awareness among oncologists, facilitating timely diagnosis and treatment.

## 2 Case

**FIGURE 1:**
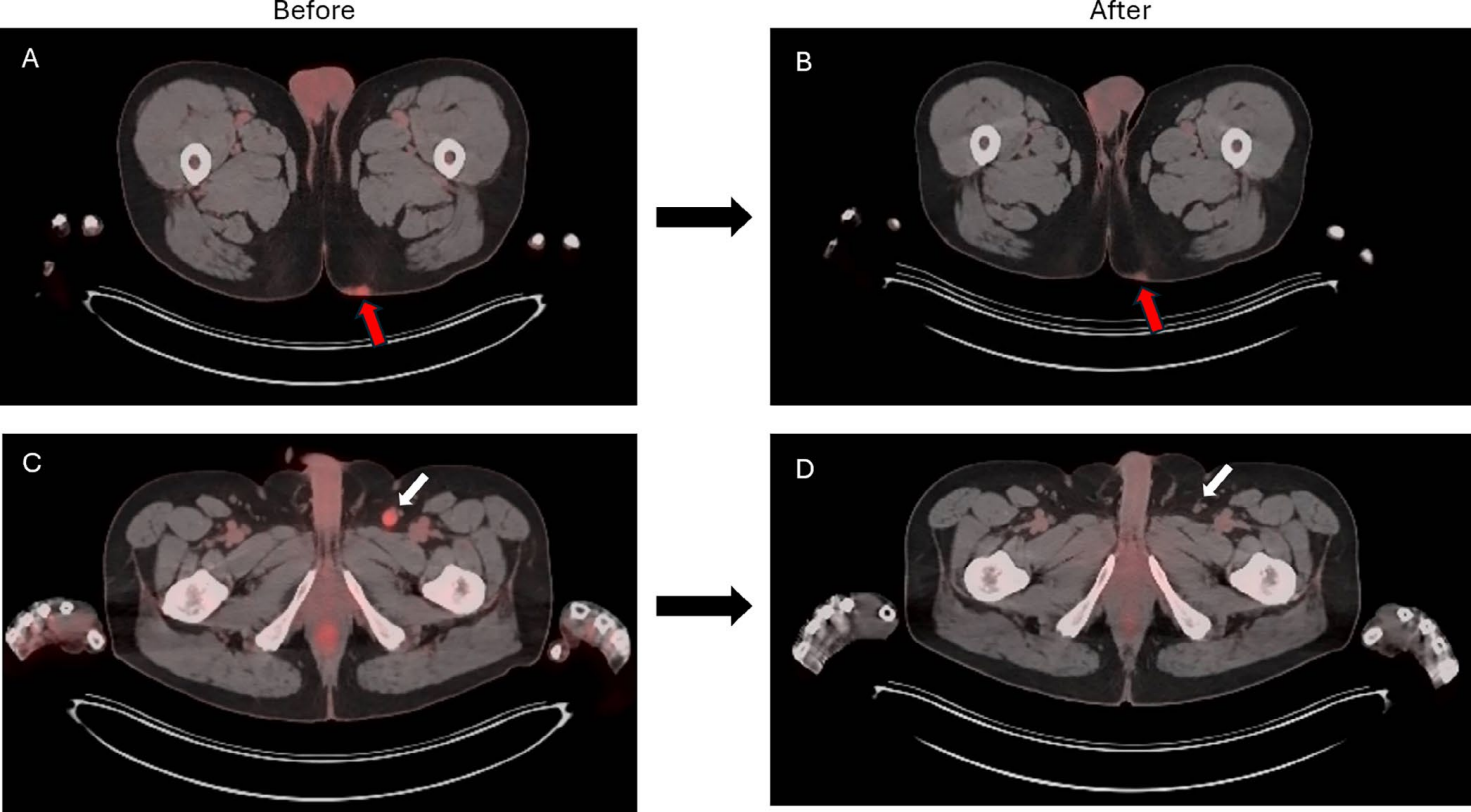
Positron emission tomography (PET) of patient's pelvic cavity taken before initiation of and after two cycles of neoadjuvant nivolumab. (A) PET scan taken before neoadjuvant nivolumab shows hypermetabolic thickening in the left gluteal cutaneous and subcutaneous soft tissues, representing known Merkel cell carcinoma (red arrow). (B) PET scan taken in February 2024 after two cycles of neoadjuvant nivolumab shows decreased degree of left gluteal skin thickening and uptake (red arrow). (C) Same PET scan as (A), showing an enlarged, hypermetabolic left inguinal lymph node (white arrow), may indicate metastatic disease without evidence of distant metastasis. (D) Same PET scan as (B), showing decreased uptake in previously hypermetabolic left inguinal lymph node (white arrow) compared to initial PET scan, compatible with favorable treatment response.

A 57‐year‐old male patient with a past medical history of mild hearing loss, nausea on 8 mg ondansetron every 8 h as needed, gastroesophageal reflux disease (GERD) on 20 mg pantoprazole daily, leishmaniasis, post‐traumatic stress disorder, prostate cancer, increased urinary frequency on 0.4 mg tamsulosin daily, and hyperlipidemia on 10 mg rosuvastatin nightly, presented to his care provider at the University of Virginia Health System in October 2023 with a left buttock mass and discomfort. The mass was biopsied and histopathological exam disclosed features of high‐grade neuroendocrine carcinoma with positive cluster of differentiation (CD) 56, cytokeratin (CK) 8/anti‐cytokeratin (CAM) 5.2, and CK20 immunostaining, confirming a diagnosis of MCC. The patient underwent subsequent PET imaging for staging, which demonstrated avid disease in the left inguinal lymph node and buttock (Figure 1). Fine needle aspiration of a left inguinal lymph node also revealed MCC. A multidisciplinary discussion favored the use of neoadjuvant nivolumab therapy.

The patient underwent his first 480 mg neoadjuvant nivolumab in 50 mL 0.9% sodium chloride infusion in late January 2024, which he tolerated, but did complain of intermittent loose stools, appetite loss, and unintended weight loss. In late February 2024, he received his second cycle of nivolumab and began experiencing increasing nausea, causing him to miss his subsequent infusion appointment. In mid‐March 2024, he presented to his local emergency department after sustaining a fall with associated nausea, emesis, and dehydration and was discharged with an indwelling urinary catheter for urinary retention. At a follow‐up visit, the patient reported hearing loss in his left ear, orthostatic hypotension resulting in loss of consciousness, urinary retention, and binocular diplopia with intermittent exotropia. Differential diagnoses included metastatic disease, autoimmune adrenal insufficiency, paraneoplastic syndromes, and dysautonomia secondary to immunetherapy toxicity. With this differential in mind, the patient was admitted for further evaluation. During admission, the patient's comprehensive metabolic panel and cortisol studies returned within normal limits. His evaluation was otherwise unrevealing for metastatic disease. Given the severity and persistence of his symptoms, atypical irAE was considered. He was ultimately diagnosed with ICI‐related dysautonomia (Table 1). The patient was treated with empiric prednisone at 1 mg/kg and with the addition of midodrine. He was stabilized and discharged with instructions on self‐catheterization due to ongoing, but improving, urinary retention.

**TABLE 1:** Patient's non‐revealing endocrine workup from March 2024 confirming the ICI‐related dysautonomia diagnosis.

Tests	Patient values	Reference ranges
Na^+^ (mEq/L)	137	135–145
Baseline cortisol (μg/dL)	16.2	10–20
Cortisol 30 min after ACTH stimulation test (μg/dL)	30.5	> 18–20
Cortisol 60 min after ACTH stimulation test (μg/dL)	32.5	> 18–20
ACTH (pg/mL)	11	7–63
LH (mIU/mL)	1.64	1.8–8.6
FSH (mIU/mL)	2.47	1.4–15.4
Prolactin (ng/mL)	16.6	2–18
TSH (mIU/L)	1.27	0.45–4.50

Two weeks later, the patient was readmitted after presenting to clinic with continued urinary retention lacking improvement, marked hypotension, and ICI‐related hepatitis. Further infectious, endocrine, and neurologic workup and a paraneoplastic panel were unrevealing. Due to initial concern for autoimmune encephalitis and malignancy, the patient underwent MRI of the brain and spine which revealed no acute abnormalities. Liver ultrasound, ordered due to the patient's newly elevated liver enzymes, showed no signs of cirrhosis or malignancy. However, it did show increased echogenicity, commonly seen with hepatocellular dysfunction.

The patient was treated with 1 g methylprednisolone daily for 5 days and was discharged on prednisone 60 mg daily with outpatient taper and 5 mg midodrine three times daily. On outpatient follow‐up, the patient continued to experience intermittent orthostatic hypotension with resolved ICI‐related hepatitis and urinary retention. The patient was admitted in late May 2024 for five doses of 0.4 g/kg intravenous immunoglobulin (IVIG) treatment, which he tolerated without any adverse effects. By June 2024, the patient was able to fully taper off his prednisone without any difficulties and continued taking midodrine, with slow improvements in his orthostatic hypotension. During this time period, the patient also developed a left‐arm melanoma.

**FIGURE 2:**
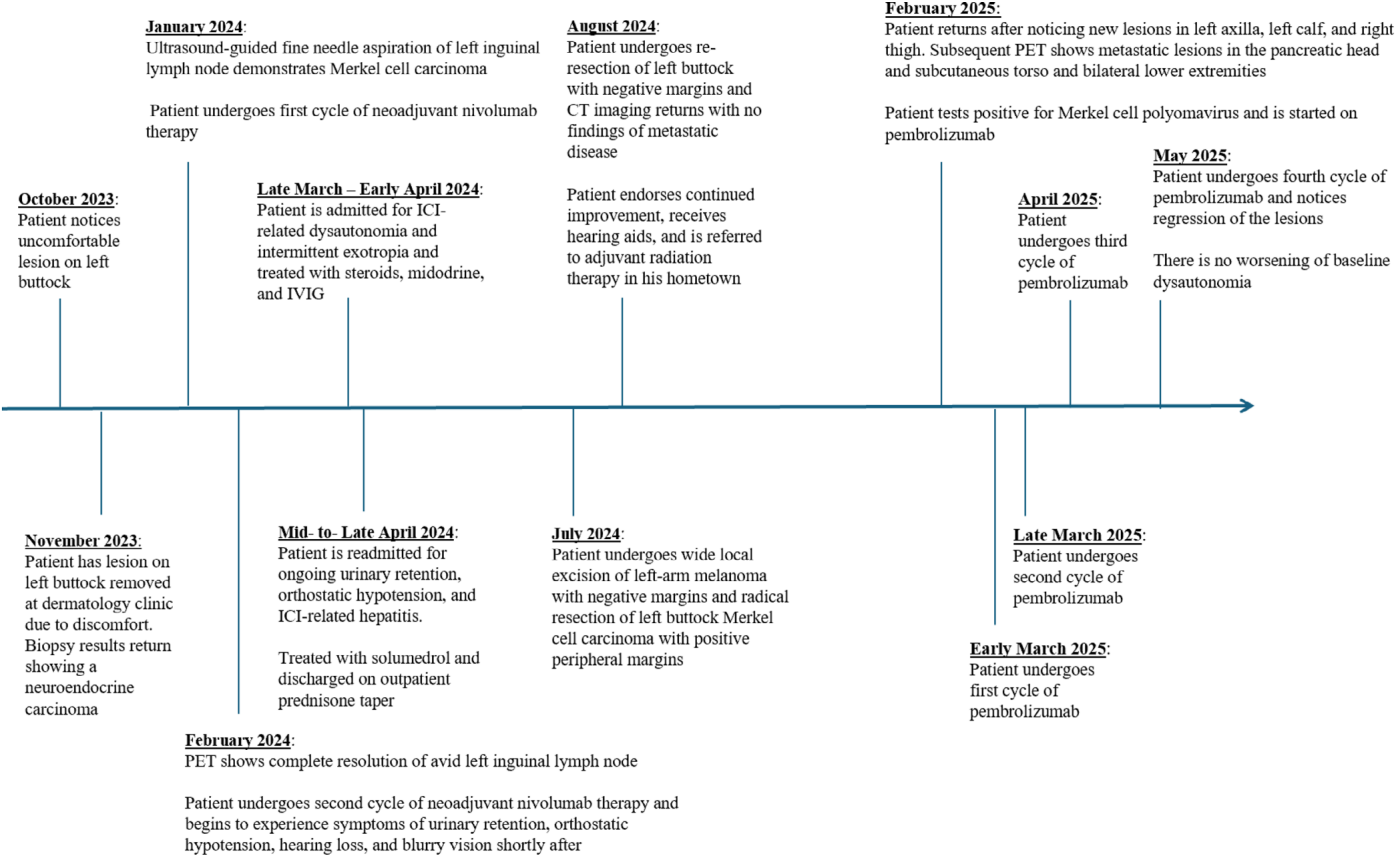
Timeline of patient's course from October 2023 to May 2025.

In late July, the patient underwent wide local excision of his left‐arm melanoma primary site and radical resection of his left buttock for MCC. No malignant melanoma was noted in his left arm, but pathology of the patient's left buttock demonstrated residual MCC with positive peripheral margin. The patient had complete resolution of inguinal lymphadenopathy. Given his complex clinical course, the decision was made to proceed with a second resection of the left buttock without therapeutic lymph node dissection, opting instead for definitive radiation to the lymph node basin. He underwent re‐resection of his left buttock in early August 2024, which returned negative for residual MCC. Repeat CT imaging showed no metastatic disease (Figure 2). During this period, the patient reported improvement of his symptoms with aggressive physical therapy and discontinuation of immunotherapy but continued to require midodrine for ongoing orthostatic hypotension. By late August 2024, the patient endorsed continued improvement since discharge, noting decreased episodes of orthostatic hypotension and benefit from receiving hearing aids. The patient has completed a course of adjuvant radiation to the left buttock primary site and left inguinal nodal basin.

He returned in February 2025 after noticing new lesions in his left axilla, left calf, and right thigh. Subsequent PET scan showed evidence of metastatic lesions in the pancreatic head and subcutaneous torso and bilateral lower extremities. He tested positive for Merkel cell polyomavirus and was started on 200 mg pembrolizumab in 50 mL 0.9% sodium chloride infusion. Thus far, he has undergone four cycles of pembrolizumab and noticed regression of the subcutaneous lesions. The patient is due for a PET scan after this fourth cycle of pembrolizumab. He has not experienced worsening of baseline dysautonomia symptoms and continues to have mild urinary retention that he is managing with a urologist.

## 3 Discussion

In this case report, we describe the variety of clinical features of dysautonomia related to ICI treatment, including orthostatic hypotension, urinary retention, hearing loss, and binocular diplopia. Dysautonomia developed after our patient's second cycle of nivolumab treatment, consistent with previous case reports detailing dysautonomia onset within 2 months of initial ICI treatment [10]. The dysautonomia was presumed an irAE linked to nivolumab and was treated with steroids, midodrine, and IVIG, resulting in overall improvement. This case report demonstrates the importance of assessing the overall clinical picture in the setting of presumed irAEs to allow for timely treatment.

ICIs target the immune checkpoint protein receptors cytotoxic T‐lymphocyte antigen (CTLA)‐4 and programmed death (PD)‐1 as well as PD‐1 ligand (PDL‐1) and lymphocytes activation gene (LAG)‐3 [7, 12]. The development of irAEs is not completely understood but several mechanisms have been proposed including receptor loss of function in these pathways [8]. In theory, blocking immune checkpoint receptors may impact self‐tolerance, resulting in a T‐lymphocyte‐dependent activation of B‐lymphocytes and subsequent development of a wide range of autoimmune pathologies [13]. Additionally, blocking CTLA‐4 may impair T‐lymphocyte and macrophage recognition of and response to antigens, resulting in endoneurial vascular inflammation and ultimately, peripheral neuropathy [14]. Future research should focus on irAE pathogenesis mechanisms and the immune components involved.

Risk factors for developing irAEs include underlying autoimmune diseases, younger age, and female sex [15, 16]. It is important to note that while younger patients are at increased risk for developing severe irAEs, older patients were found to have lengthier hospital stays and increased death rates [15]. At the onset of the dysautonomia, the patient was 57 years old, increasing his risks for longer hospital stays and mortality. Prior to the development of the ICI‐related dysautonomia, the patient had chronic nausea, mild hearing loss, and urinary frequency; however, he did not have any underlying autoimmune diseases or hepatitis. The patient was taking rosuvastatin, escitalopram, pantoprazole, ondansetron, and tamsulosin. The patient's liver enzymes were normal prior to ICI‐related hepatitis onset and his chronic nausea, mild hearing difficulties, and urinary symptoms were likely exacerbated by the ICIs. Tamsulosin was held at the first hospital admission, however, the patient continued to have refractory orthostatic hypotension. Providers also determined that the escitalopram was not a likely contributor to the patient's orthostatic hypotension, hepatitis, urinary retention, or diplopia.

Severity of irAEs is associated with class of ICI used in treatment [9]. PD‐1 is expressed on T cells after activation and inhibits T‐cell receptor signaling. PDL‐1 can be upregulated in response to inflammatory signals in both healthy and malignant cells [7]. Gastrointestinal inflammation and hypophysitis are more common with CTLA‐4 blockade, while thyroiditis, pneumonitis, and autoimmune diabetes are more commonly associated with PD‐1 blockade [8, 17, 18].

Neurological irAEs occur in approximately 1%–5% of patients treated with an ICI and can be minor such as headaches or vertigo, or more severe, involving the central and peripheral nervous systems. Central nervous system irAEs typically manifest as aseptic meningitis, encephalitis, or myelitis while peripheral nervous system irAEs manifest as myositis, cranial neuropathy, myasthenia gravis, and polyradiculopathies, and/or a variety of dysautonomias [19, 20, 21]. ICI‐induced autonomic dysfunction is a rare neurologic irAE. The autonomic nervous system is responsible for regulating bodily functions such as sweating, heart rate, respiratory rate, blood pressure, digestion, and urination. Therefore, ICI‐induced dysautonomia can affect any of these functions. The most common clinical manifestations of ICI‐induced dysautonomia are gastrointestinal disturbances and orthostatic hypotension [10].

A recent review classifies ICI‐related dysautonomia into two types: autoimmune autonomic ganglionopathy (AAG) and autonomic neuropathy [22]. AAG encompasses a variety of autonomic symptoms and can be accompanied by autoimmune encephalitis and/or autoimmune rheumatological diseases [13]. Approximately 50% of patients with AAG have autoantibodies to ganglionic nicotinic acetylcholine receptors (gAChR) [23]. However, the presence of gAChR antibodies is not necessary for an AAG diagnosis [13]. On review of our patient's diagnostic workup, a test for autoantibodies to gAChR was not performed. Completion of this test may have aided in a timelier diagnosis and treatment for our patient.

Autonomic neuropathy is a subset of motor and sensory neuropathies specifically affecting autonomic fibers [10]. These broad definitions of AAG and autonomic neuropathy make distinguishing between other irAEs and ICI‐related dysautonomia challenging. This diagnostic dilemma is further complicated by the potential co‐occurrence of irAEs with ICI‐related dysautonomia, such as concurrent ICI‐related hepatitis in our patient.

Treatment varies by irAE grade. Based on the 2022 European Society for Medical Oncology (ESMO) Clinical Practice Guideline for irAEs, corticosteroids are the first‐line therapy for irAEs with dose corresponding to irAE grade. ICI therapy should continue for grade 1 irAEs, except in specific cases of cardiac and/or neurological toxicities. ICI therapy should be withheld in grade 2 irAEs, and patients should receive prednisolone 0.5–1 mg/kg/day. Patients with grade 3 irAEs should be admitted and given prednisolone or methylprednisolone 1–2 mg/kg/day. Corticosteroid treatment can be tapered if the patient displays 48 h of continuous symptom improvement. If the patient is experiencing irAEs of high severity, a slow 4‐ to 6‐week taper is recommended [24]. Corticosteroid‐resistant irAEs may be treated with plasma exchange, IVIG, mycophenolate mofetil, infliximab, vedolizumab, abatacept, anti‐thymocyte globulin, and/or ruxolitinib depending on irAE type and severity [25, 26, 27].

Out of a total of 1330 irAE cases documented on the Side Effect Registry Immuno‐Oncology in 2023, IVIG was the second most utilized therapy for steroid‐resistant irAEs [28]. In addition to corticosteroid‐resistant irAEs, IVIG was effective in treating cases of ICI‐induced neutropenia, thrombocytopenia, transverse myelitis, myasthenia gravis, cranial neuropathy, vertigo, psychiatric disturbances, autoimmune encephalitis, myocarditis, AAG, Steven Johnson Syndrome/Toxic Epidermal Necrolysis, and scleroderma‐like syndrome [13, 29, 30]. Given this supporting evidence for IVIG use in ICI‐induced dysautonomia, we chose to treat our patient's unresolved orthostatic hypotension and urinary retention with IVIG, which proved effective. Therefore, it may be beneficial to administer IVIG during the course of ICI‐related dysautonomia.

Despite the growing number of ICI‐related dysautonomia cases reported in the literature, its pathogenesis is still poorly understood, and management remains undefined. Previously published cases of anti‐PD‐1 blockades discuss the development of primarily neuromuscular complications, including myasthenia gravis and necrotizing myopathy [31, 32, 33, 34]. However, in our case, we discuss dysautonomia caused by an anti‐PD‐1, resulting in orthostatic hypotension, urinary retention, diplopia, as well as hepatitis. Additionally, our patient did not have an underlying autoimmune condition or hepatitis that may have predisposed him to develop ICI‐related dysautonomia and immune‐mediated hepatitis respectively [35, 36, 37]. Past and ongoing ICI clinical trials exclude patients with underlying autoimmune conditions. Future research should focus on the efficacy of ICIs in patients with underlying autoimmune disorders as well as any irAEs that may develop in the treatment course.

Screening protocols for ICI‐related dysautonomia will aid in early detection and management. However, as ICI‐related dysautonomia is a rare entity, screening should only be completed if there is clinical concern for it. Diagnostic workup should include a comprehensive metabolic panel, cortisol studies, thyroid studies, cerebrospinal fluid studies, a paraneoplastic panel, imaging of the brain and spine to rule out underlying malignancy, test for antibodies to gAChR, as well as any additional imaging based on workup results and symptoms, such as a liver ultrasound in our patient with elevated liver enzymes and signs of hepatitis. Treatment options include corticosteroid treatment based on ESMO guidelines, midodrine for orthostatic hypotension, and IVIG.

New onset dysautonomia without predisposing conditions and/or worsening of underlying conditions, such as chronic nausea, hearing loss, and urinary retention in our patient, in the setting of recent ICI treatment should raise concern for ICI‐related dysautonomia. If there is a concern for ICI‐related dysautonomia, undergoing workup based on our suggested screening protocol may aid in timely diagnosis and intervention.

In conclusion, we report a case of ICI‐related dysautonomia with concurrent ICI‐related hepatitis, requiring steroid, midodrine, and IVIG therapy. Despite the rarity of this irAE and mixed responses to suggested first‐line therapies, oncologists should be aware of dysautonomia associated with ICI use, especially in the setting of co‐occurring irAEs. Future directions may include research into the types of ICIs resulting in dysautonomia and the potential pathways involved, in order to aid in developing more effective treatment options.

## Author Contributions

**Nidhi Kuchimanchi:** writing – original draft. **Sai Gajula:** writing – original draft. **Elizabeth M. Gaughan:** writing – review and editing. **Russell G. Witt:** conceptualization, writing – review and editing, supervision.

## Consent

Written informed consent was obtained from the patient for the publication of case details and use of images.

## Conflicts of Interest

The authors declare no conflicts of interest.

## Data Availability

Data sharing is not applicable to this article as no new data were created or analyzed in this study.
